# Expression pattern of arenicins—the antimicrobial peptides of polychaete *Arenicola marina*

**DOI:** 10.3389/fphys.2014.00497

**Published:** 2014-12-19

**Authors:** Arina L. Maltseva, Olga N. Kotenko, Vladimir N. Kokryakov, Viktor V. Starunov, Anna D. Krasnodembskaya

**Affiliations:** ^1^Department of Invertebrate Zoology, Saint Petersburg State UniversitySaint Petersburg, Russia; ^2^Department of Biochemistry, Saint Petersburg State UniversitySaint Petersburg, Russia; ^3^Department of General Pathology and Pathophysiology, Institute of Experimental Medicine, Russian Academy of Medical SciencesSaint Petersburg, Russia; ^4^Centre for Infection and Immunity, School of Medicine, Dentistry and Biomedical Sciences, Queens University BelfastUK

**Keywords:** antimicrobial peptides, invertebrate immunity, coelomocytes, *Arenicola marina*, annelid

## Abstract

Immune responses of invertebrate animals are mediated through innate mechanisms, among which production of antimicrobial peptides play an important role. Although evolutionary Polychaetes represent an interesting group closely related to a putative common ancestor of other coelomates, their immune mechanisms still remain scarcely investigated. Previously our group has identified arenicins—new antimicrobial peptides of the lugworm *Arenicola marina*, since then these peptides were thoroughly characterized in terms of their structure and inhibitory potential. In the present study we addressed the question of the physiological functions of arenicins in the lugworm body. Using molecular and immunocytochemical methods we demonstrated that arencins are expressed in the wide range of the lugworm tissues—coelomocytes, body wall, extravasal tissue and the gut. The expression of arenicins is constitutive and does not depend on stimulation of various infectious stimuli. Most intensively arenicins are produced by mature coelomocytes where they function as killing agents inside the phagolysosome. In the gut and the body wall epithelia arenicins are released from producing cells via secretion as they are found both inside the epithelial cells and in the contents of the cuticle. Collectively our study showed that arenicins are found in different body compartments responsible for providing a first line of defense against infections, which implies their important role as key components of both epithelial and systemic branches of host defense.

## Introduction

Antimicrobial peptides (AMPs) are relatively small (not exceeding 100 amino acids) usually cationic polypeptidic molecules with prominent inhibitory potential against various microbial pathogens. It is well recognized that AMPs constitute an important component of the innate immunity, having a role in both effector and regulatory functions. The fact that AMPs are present in a wide range of organisms including plants, vertebrate and invertebrate animals, protists and prokaryotes (rev. in Boman, 2003; Reddy et al., 2004; Yount et al., 2006; Otero-González et al., 2010; Pasupuleti et al., 2012) points to their importance for host defense against infections throughout the evolutionary process.

The wide distribution of AMPs makes them an attractive object for comparative immunology. Such studies can provide insights into the mechanisms underpinning the evolution of innate immune responses and also lead to identification of new effective molecular structures which could be developed as new therapeutics. AMPs of invertebrate animals are of particular importance, as their host defense against infections relies entirely on the mechanisms of innate immunity. Historically insects represent a group of invertebrate animals where the immune system has drawn intensive interest of investigators and consequently their AMPs are the most well characterized (Bulet et al., 1999; Otvos, 2002; Bulet and Stocklin, 2005), whereas the knowledge about AMPs from other invertebrates remains limited.

Annelids might be considered as a basal group which is closest to a putative common ancestor of other coelomate invertebrates. Therefore, their immunity is a fascinating object of study for comparative immunologists. Besides this, annelids are important players in the benthic communities as they are critical for biomass production in marine and freshwater ecosystems. Paradoxically, the existing knowledge about mechanisms of their host defense is largely based on the studies of Oligochaetes (Cooper et al., 2004; Kauschke et al., 2007; Bilej et al., 2010) or Hirudineans (Tasiemski and Salzet, 2010), which are relatively specialized branches of Annelids, whereas more basal Polychaetes still fall out of scope of investigation.

More specifically, just a few species of annelids were investigated as possible sources of AMPs (rev. in Tasiemski, 2008). Among polychaetes these are: *Perinereis aibuchitensis* (Pan et al., 2004), *Nereis diversicolor* (Tasiemski et al., 2007)—two closely related genera—and *Arenicola marina* (Ovchinnikova et al., 2004). There is no structural homology between AMPs from listed species as well as with any other published AMP. Up to date, only hedistin from *N. diversicolor* was studied in terms of its functional activity within the host. Hedistin was shown to be exclusively and constitutively expressed in so called NK cells (one of the coelomocyte types) (Tasiemski et al., 2007).

Arenicins-1 and -2, identified by our group in *A. marina*, are 21 residue peptides. They differ by one amino acid replacement and their ß-hairpin structure is supported by one disulfide bond (Ovchinnikova et al., 2004). Structural properties make them feasible for chemical and recombinant synthesis (Kolodkin et al., 2006; Ovchinnikova et al., 2007). These peptides have been extensively studied during the last decade (Lee et al., 2007; Andra et al., 2008, 2009; Park and Lee, 2009; Cho and Lee, 2011). Collectively, structural and functional properties make them promising candidates for preclinical studies. However, from the comparative immunological perspective, it is important to establish the functional role of these peptides for the host defense of the organism.

In the present study we addressed the question of the physiological functions of arenicin 1 and 2 in the lugworm body as components of its immune system with a specific focus on the spatial distribution of their localization in the tissues and the pattern of their expression upon infectious stimuli.

## Materials and methods

### Animals and tissues

#### Animals

Adult lugworms (apprx. 2.5 cm length) *Arenicola marina* individuals were collected from wild populations over the intertidal zone in the vicinity of the White Sea Marine biological station of Saint Petersburg University (Russia). Animals were maintained in permanently aerating static tanks with marine water for 5–7 days.

#### Stimulation

To induce immune responses worms were injected by 10^7^ CFU suspension of heat inactivated *Escherichia coli ML35p*, *Listeria monocytogenes EGD*, *Candida albicans 820* (alone or in combination) in 1 ml of sterile marine water (SMW), control animals received SMW; all microbial strains were kindly provided by Prof. R. Lehrer, University of California, Los Angeles, USA. Control animals were injected by equivalent volume of SMW. Coelomic fluid (CF) of 5 animals representing each group was pooled at 24 and 48 h after immunization. CF was collected from the middle part of a lungworm body with a sterile syringe (*d* = 0.6 mm) needle filled with an ice-cold SMW to prevent coagulation: CF to SMW ratio remained 1: 2. CF was sedimented at 400 g for 10 min at 4°C directly after collection. Precipitated cells were suspended in cold SMW for smear preparation or used for RNA extraction.

#### Other tissues

Before dissection animals were anesthetized in 5% MgCl_2_ in SMW whereupon pieces of body wall, salivary glands, foregut, and midgut tissue were dissected, placed into sterile tubes and fixed for RNA extraction.

### PCR

#### Template

RNA was isolated from fixed and homogenized in PureZOL (Cat. No. #732-6890, BioRad Inc, CA, USA) tissues, using the RNAeasy kit (Cat. No. 74104 Qiagen Inc., CA, USA). After DNase I (Cat. No. #EN0521 Fermentas, Thermo Fisher Scientific Inc., MA, USA) treatment (30 min at room temperature) and purification RNA quality was verified with NanoDrop® ND-1000 UV-Vis Spectrophotometer (NanoDrop Technologies, Wilmington, DE, USA). 260/280 and 260/230 nm absorbance ratios did not exceed 2.0 indicating appropriate RNA quality.

#### Semiquantative PCR

Arenicin-1, arenicin-2 and actin primers were custom made by Syntol C° (Moscow, Russia). The sequences were as follows.

Arenicin-1: forward 5′-CTAATCCTGGCCATTTTCTGCG-3′; reverse 5′-CCCTGAGCTGACTGGAAATAG-3′; product 338 bp.

Arenicin-2: forward 5′-GCGAGATCGGCTGGAGAG-3′; reverse 5′-CCCTGAGCTGACCGGAAG-3′; product 254 bp.

Actin: forward 5′-CAAATCATGTTCGAGACCTTC-3′; reverse 5′-GCTGATCCACATCTGTTGG3′; product 714 bp.

PCR was performed with SuperScript™ III One-Step RT-PCR System with Platinum® Taq DNA Polymerase protocol (Cat. No. 12574-018, Invitrogen, Thermo Fisher Scientific Inc., MA, USA) according to manufacturer's instructions. β-Actin gene was used as a control to normalize a template loading. Amplification was carried out at C1000X cycler (BioRad Inc., CA, USA). The PCR products were then sequenced in Resource Center for Molecular and Cellular Technologies of St. Petersburg State University (St.Petersburg, Russia).

#### qRT-PCR

Real-time PCR was performed with iQ™ SYBR® Green Supermix (Cat. No. 170-8880, BioRad, CA, USA). Five independent RNA isolations with following cDNA synthesis and RT PCR were done for each experimental case. Reactions were conducted on CFX100 cycler (BioRad, CA, USA). Actin was used as a housekeeping gene for normalization and results were analyzed by the ΔCT method to calculate expression changes, *P* < 0.05 was considered to be statistically significant.

### Antibody

Recombinant arenicin-2 (Ovchinnikova et al., 2007) was conjugated with cargo-protein and used to obtain polyclonal antiserum. The conjugate was compounded with complete Freund adjuvant (Cat. No. F5881, Sigma-Aldrich, MO, USA) and injected into rabbit males at a dose 1 μg per gram. The same dose was used for secondary injection 2 weeks later. Serum samples were collected three times—4, 6, and 8 weeks after first immunization. The titer of reactive antibody in the serum samples was examined by immunoblotting against natural (in crude extract) and synthetic arenicin-1 (Kolodkin et al., 2006). Active samples were pooled and stored at 4°C. Specific anti-arenicin antibody was purified from the serum by affine chromatography using CNBr-activated Sepharose (Cat. No. GE17-0430-01 Sigma-Aldrich, MO, USA) conjugated with recombinant arenicin-2. Anti-arenicin AB was eluted by NaCl gradient and desalted on Amicon Ultra centrifugal filters Ultracel 3K (Cat. No. Z677094-24EA, Millipore, Merck KGaA, Germany). AB specificity was tested by immunoblotting against natural (in the crude extract) and synthetic arenicin-1 (Kolodkin et al., 2006).

### Immunoblotting

Crude extracts of coelomocytes were obtained by homogenization of coelomocytes (prepared as described earlier) in 5% acetic acid. Particles were sedimented by centrifugation (10,000 g, 40 min, 4°C) and supernatants were aspirated and accepted as crude extracts. Synthetic arenicin-1 was kindly provided by Dr. Nickolai I. Kolodkin (Institute of Highly Pure Biopreparations, Saint Petersburg, Russia). Both crude extract (50 μg of total protein) and arenicin-1 (0.5 μg) were loaded into acidic urea 12.5% PAAG (Panyim and Chalkley, 1969) and transferred onto PVDF immune blot membrane (Cat. No. 162-0177, BioRad Inc., CA, USA) in TetraCell Miniprotean Chamber (BioRad Inc., CA, USA) under standard conditions. The membrane was equilibrated in PBST (3 times, 10 min), blocked by bovine serum albumin (Cat. No. A2153, Sigma-Aldrich, MO, USA) overnight and stained initially by polyclonal serum or purified anti-arenicin AB (1 h) and further 1 h by secondary sheep HRP-conjugated anti-rabbit AB (Cat. No. #31463, Thermo Scientific, Thermo Fisher Scientific Inc., MA, USA). DAB (Cat. No. D8001, Sigma-Aldrich, MO, USA) was used for visualization.

### Immunocytochemistry

#### Cross-sections

Lugworms (apprx. 2.5 cm length) were anesthetized in 5% MgCl_2_ in SMW, fixed in 4% paraformaldehyde (Cat. No. 158127, Sigma-Aldrich, MO, USA), subjected to standard alcohol dehydration and via xylol embedded into paraffin. Serial cross-sections (4 μm) were placed on polylysine-coated slides (Cat. No. P0425, Sigma-Aldrich, MO, USA) and dried at 37°C.

#### Coelomocytes smears

Fresh coelomocytes in suspension were placed on polylysine-coated slides (Cat. No. P0425, Sigma-Aldrich, MO, USA), left to dry out at room temperature and fixed with 4% paraformaldehyde (Cat. No. 158127, Sigma-Aldrich, MO, USA). For *in vitro* phagocytosis, fresh coelomocyte suspension was placed into the plastic plate for 10 min to adhere in the humid chamber at 4°C. The suspension of zymosan particles (Cat. No. Z4250, Sigma-Aldrich, MO, USA) was added (MIO 10: 1) and after incubation for 15 min the preparations were washed twice with SMW and fixed in 4% paraformaldehyde.

#### Antibody staining

Prepared cross-sections or smears were incubated sequentially in PBST (3 times, 20 min), PBST with diluted sheep serum 1: 5 (1 h) and anti-arenicin AB (1: 50) in sheep-serum or rabbit IgG overnight. Preparations were washed by PBST (3 times, 20 min) and stained by HRP-conjugated secondary AB (1: 500) (Cat. No. #31463, Thermo Scientific, Thermo Fisher Scientific Inc., MA, USA). AB was visualized with DAB (Cat. No. D8001, Sigma-Aldrich, MO, USA). Some preparations were additionally stained by hematoxylin-eosin.

## Coelomic fluid plasma testing

CF was obtained as described earlier. Plasma was separated from cells by centrifugation (400 g, 10 min, 4°C). For component analysis of plasma it was loaded onto HPLC analytical column (C18, D 4.6 mm, length 20 cm, grain 5 μm, pore 300 Å, Vydec, The Nest Group Inc., MA, USA) and separated using Agilent 1260 HPLC system (Agilent Technologies, CA, USA). Elution was performed with a gradient of 0–60% acetonitrile in presence of 0.05% trifluoroacetic acid during 1 h. Absorbance at 220 and 280 nm was used for detection.

For antimicrobial testing native plasma was concentrated (3:1) in VacSpeed System (Labconco, MO, USA). Two variations of test were applied as described in Lehrer et al. (1989). In the overlay test the sample was first separated in the acidic urea PAAG by electrophoresis and then PAAG was laid over 1% agarose gel (Cat. No. A0169, Type I, Sigma-Aldrich, MO, USA) with embedded microorganisms. In the radial diffusion test the whole probe was put into the well of 1% agarose gel with immobilized microorganisms as above (4 × 10^6^ CFU per plate). After 2.5 h of diffusion, growth medium (Tryptic Soy Broth for bacteria, Cat. No. M011, or Sabauraud medium for fungi, Cat. No. M013, HiMedia Laboratories, Mumbai, India) was added and plates were incubated overnight.

## Results

### Arenicins mRNA is ubiquitously expressed in the lugworm tissues

**Figure 1:**
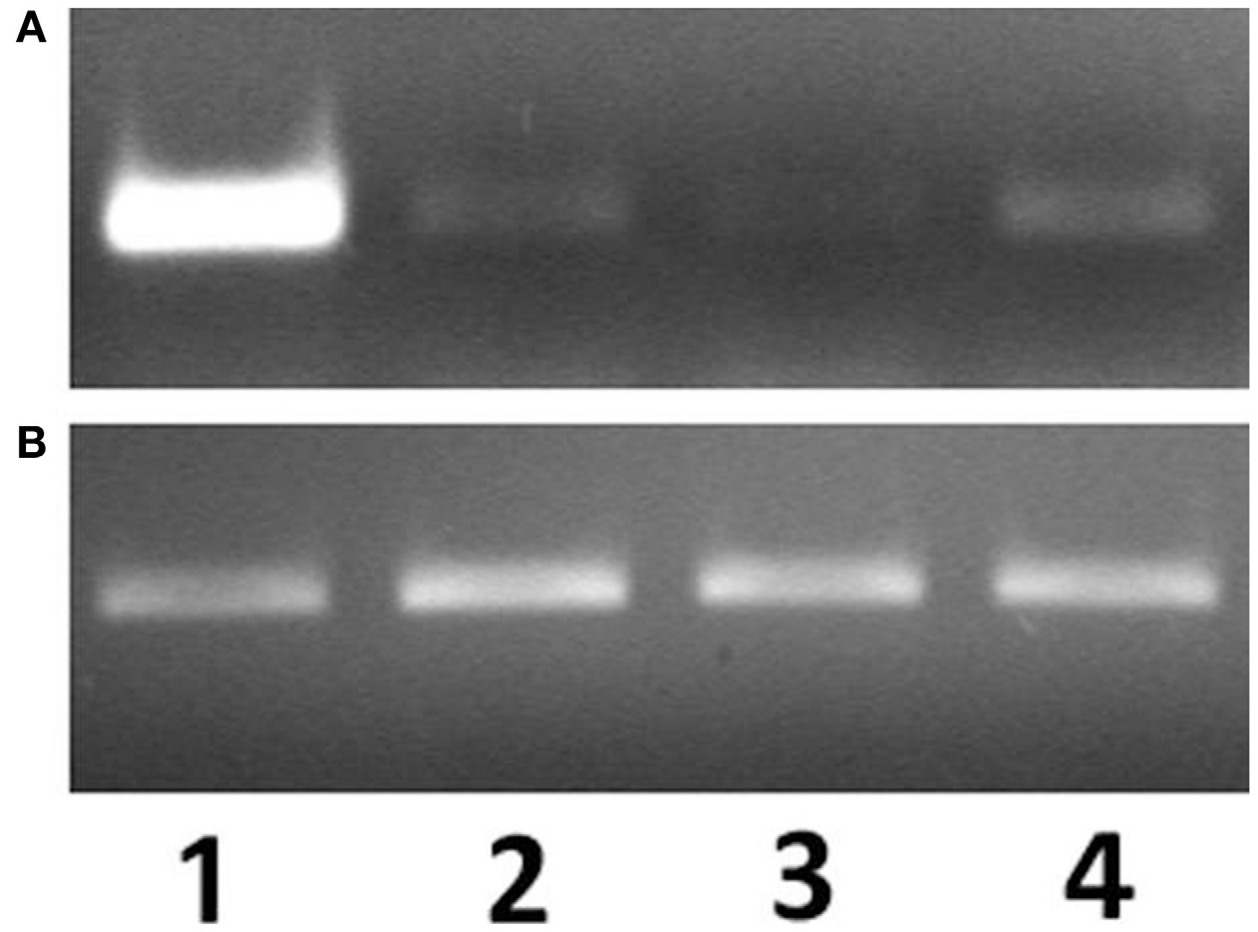
**mRNA expression levels of arenicin-1 (A) relatively to actin (B) measured by PCR**. Expression levels in coelomocytes (1), body wall (2), pharynx (3), intestine (4).

The presence of arenicins-1 and -2 transcripts were examined in coelomocytes, body wall, salivary glands, foregut, and midgut by semi-quantitative RT-PCR. Arenicins mRNA expression was detected in all types of analyzed samples, however the highest levels of both arenicin-1 and 2 mRNA were observed in coelomocytes (Figure 1; signal of arenicin-2 was markedly attenuated in all samples, Figure S1). Detection of arenicin transcripts in several different compartments of the worm body suggests their ubiquitous expression and potential involvement in different types of host defense—local or systemic. The next step was to test if their expression was sensitive to microbial invasion.

### mRNA expression of arenicins does not depend on the microbial stimulation

**Figure 2:**
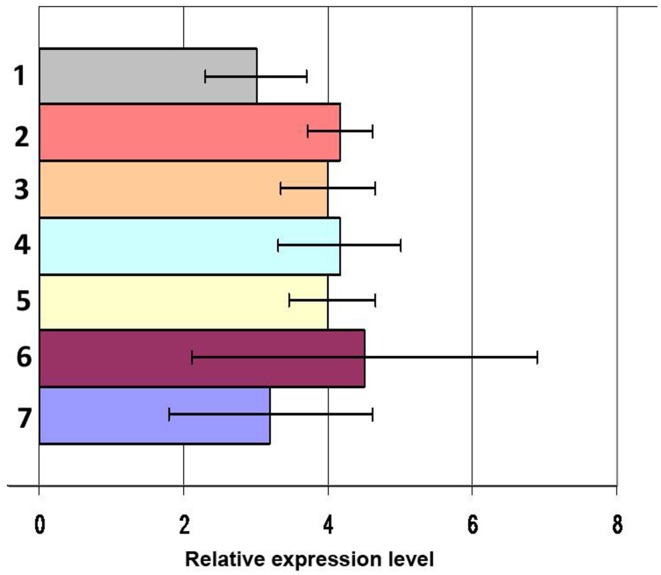
**mRNA expression levels of arenicin-1 measured by RT-PCR relatively to actin in coelomocytes after immunization**. Microbial mixture 24 h post infection (1), *C. albicans* 24 h post infection (2), *E. coli* 48 h (3) and 24 h post infection (4), *L. monocytogenes* 24 h post infection (5), PBS 24 h post infection (6), intact animals (7). Data are expressed as mean ± *SD*.

To mimic the infectious process worms were injected with heat-inactivated laboratory strains of microorganisms into the coelomic cavity. In order to investigate the possible role of different PAMPs/PRRs interaction in mediating the response, worms were challenged with representatives of the three major different lineages of microorganisms (gram-positive and gram-negative bacteria and fungi) in different combinations. At 24 h post microbial challenge, samples of different tissues were collected and arenicins mRNA expression was quantified by Real-Time PCR. There was no change in the levels of arenicin-1 and -2 expression post-stimulation of coelomocytes compared to intact or SMW-injected animals (Figure 2). Similarly, injection of heat-inactivated microbes into the coelomic cavity did not induce changes in arencins mRNA expression in the gut or body wall, compared to the controls (Figure S2). These results demonstrate that expression of arenicin-1 and arenicin-2 is constitutive and does not depend either on the nature of the pathogen or on the presence/absence of pathogens in coelomic cavity.

### Immunohistochemistry confirms ubiquitous expression of arenicins in the lugworm tissues

**Figure 3:**
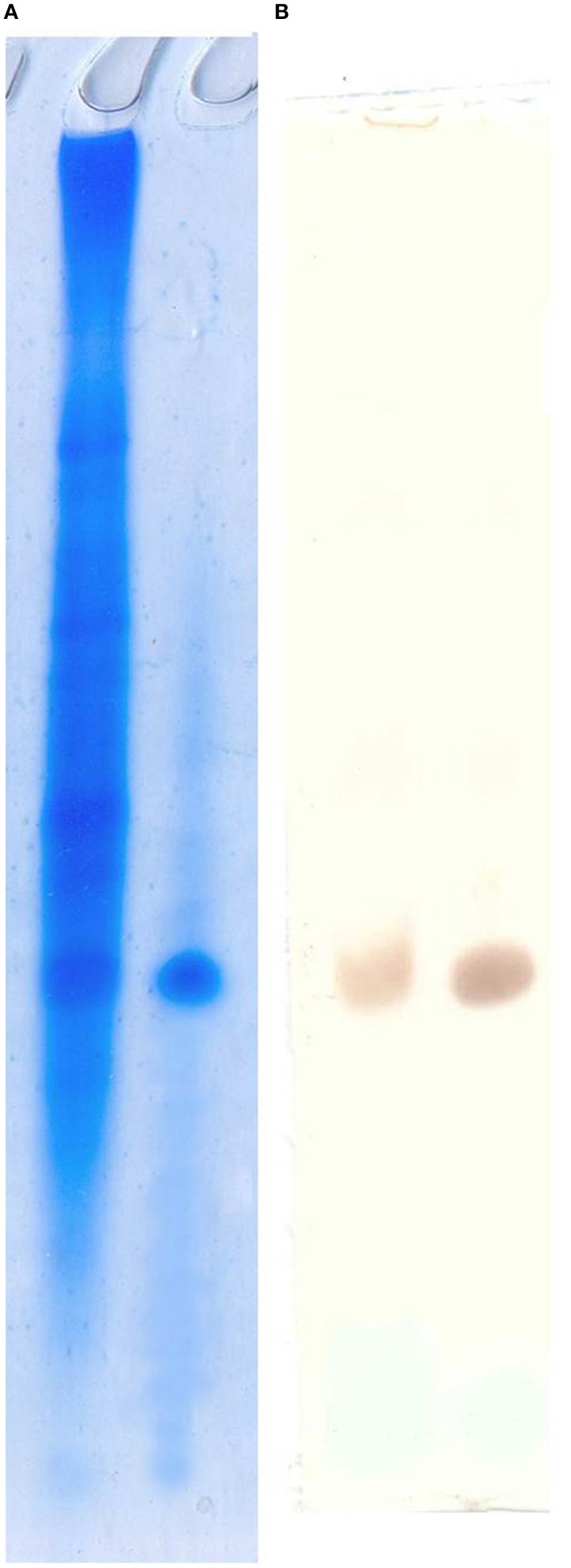
**Polyclonal antibody specificity testing by Western blot**. Acidic urea PAAG **(A)** and pAB staining visualized by DAB **(B)**. Left line, crude extract; right line, synthetic arenicin-1.

Anti-arenicins rabbit pAB were purified by affine chromatography against recombinant arenicin-2. Their specificity was confirmed by Western-blot against crude coelomocyte extract, synthetic arenicin-1 being a positive control (Figure 3). Western blotting demonstrated that pAB was specific for the mature arenicins fraction of the extract, but lacked the ability to discriminate between two isoforms of arenicin. After testing, the pAB were used for immunohistochemical analysis of lugworm tissues.

**Figure 4:**
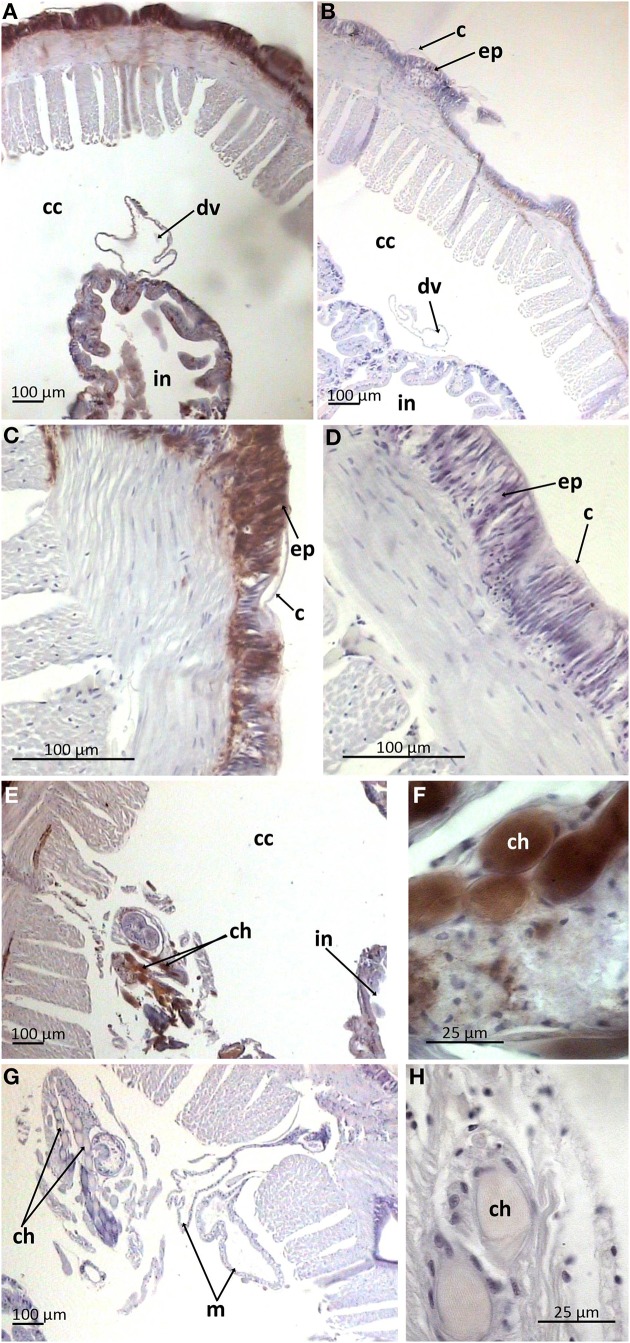
**Arenicin-positive immunoreactivity of lugworm body wall**. **A,B**, General view of dorsal body part; **C,D**, body wall; **E,F**, chaetae complex; **B,D,G,H**, negative controls; c, cuticle; cc, coelomic cavity; ch, chaetae; ep, body wall epithelium; in, intestine; m, metanephridia.

**Figure 5:**
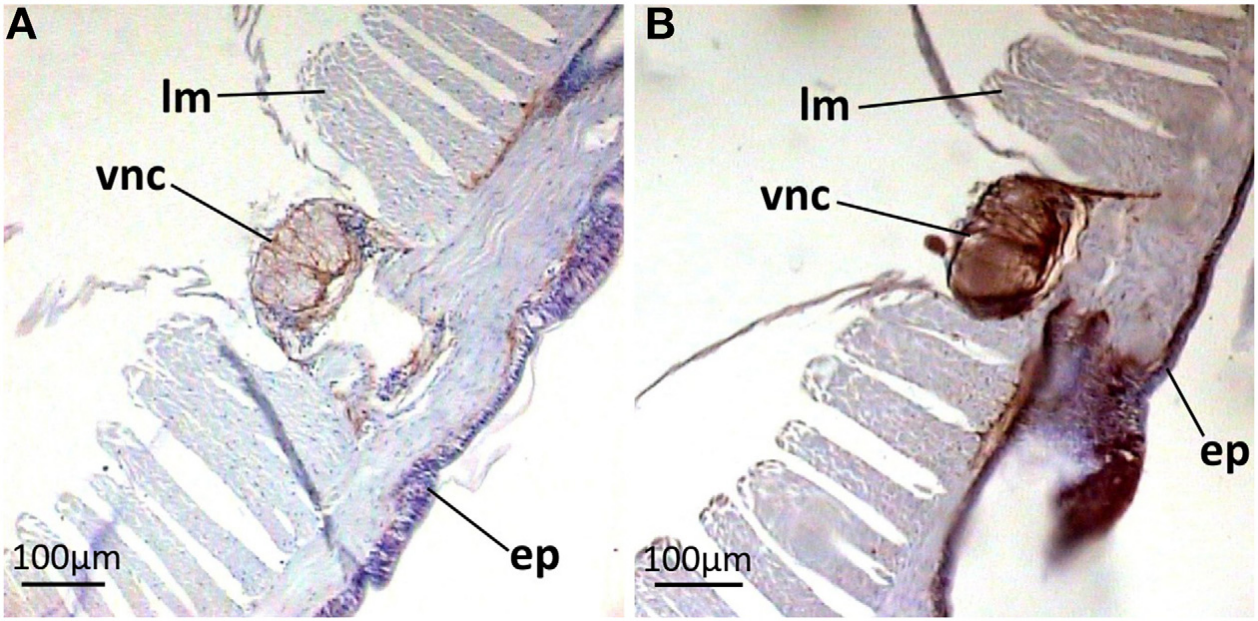
**Arenicin-positive immunoreactivity of lugworm nervous system**. **A,B**, sector of dorsal body part; **A**, negative control; ep, body wall epithelium; lm, longitudinal musculature; vcn, ventral nervous chain.

Paraffin-embedded tissue cross-sections were pAB stained to characterize mature peptide localization. Several compartments including the outermost part of body wall and gut wall proved to be pAB-immunoreactive (Figures 4A,B), which correlated with the PCR result. The arenicin-positive part of the body wall is continuous and represented by a thin cuticle and underlying epithelium lining. Unexpectedly the arenicin signal came from chaetae complex (Figures 4C–F). In addition there was evident arenicin-positive staining, associated with ventral nervous chain (even some additional background staining was also noted Figure 5).

**Figure 6:**
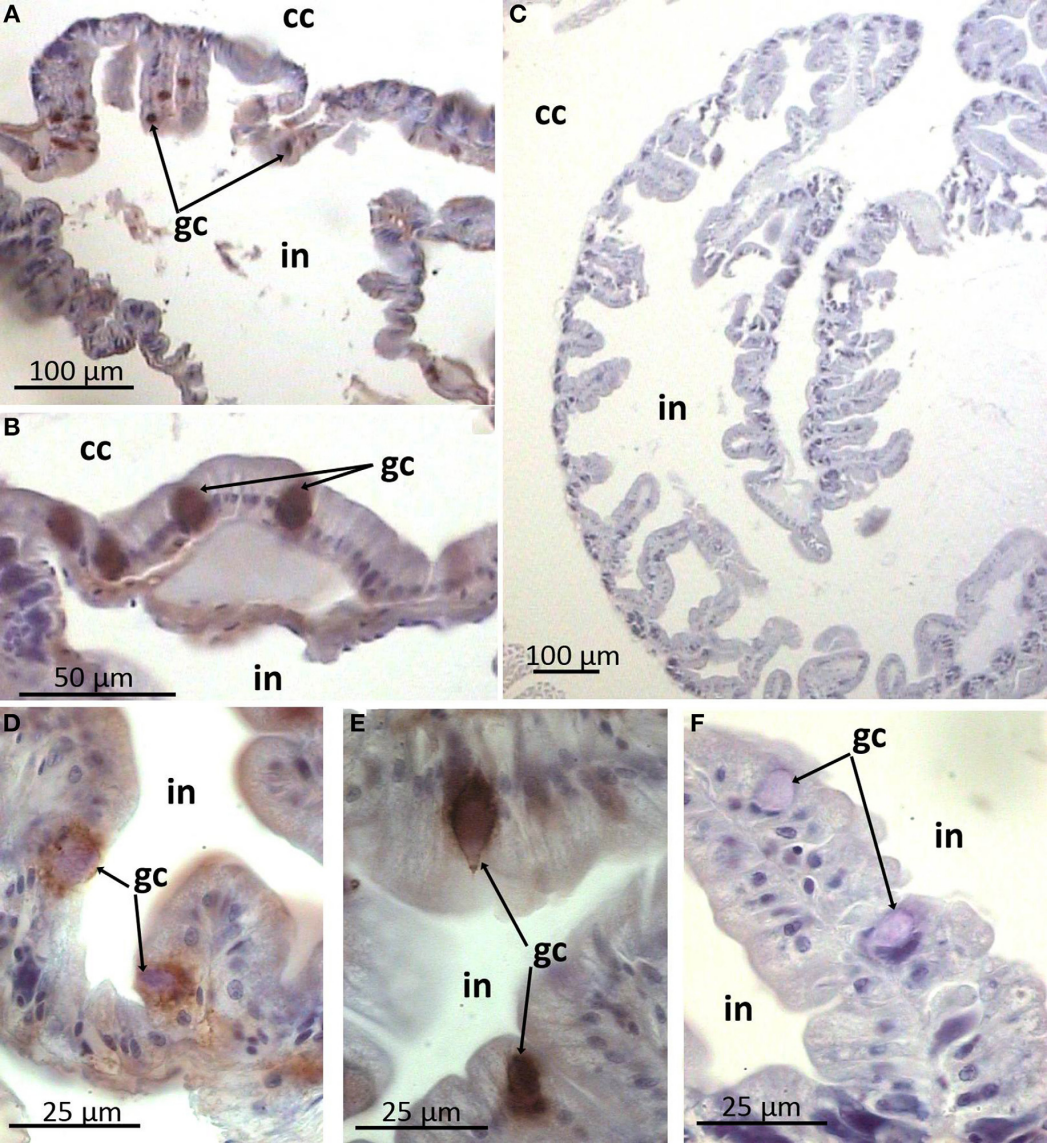
**Arenicin-positive immunoreactivity of lugworm intestine**. **A,C**, general view of intestine; **B,D–F**: sparse immunopositive cells; **C,F**, negative controls; cc, coelomic cavity; gc, presumable gland cells; in, intestine.

**Figure 7:**
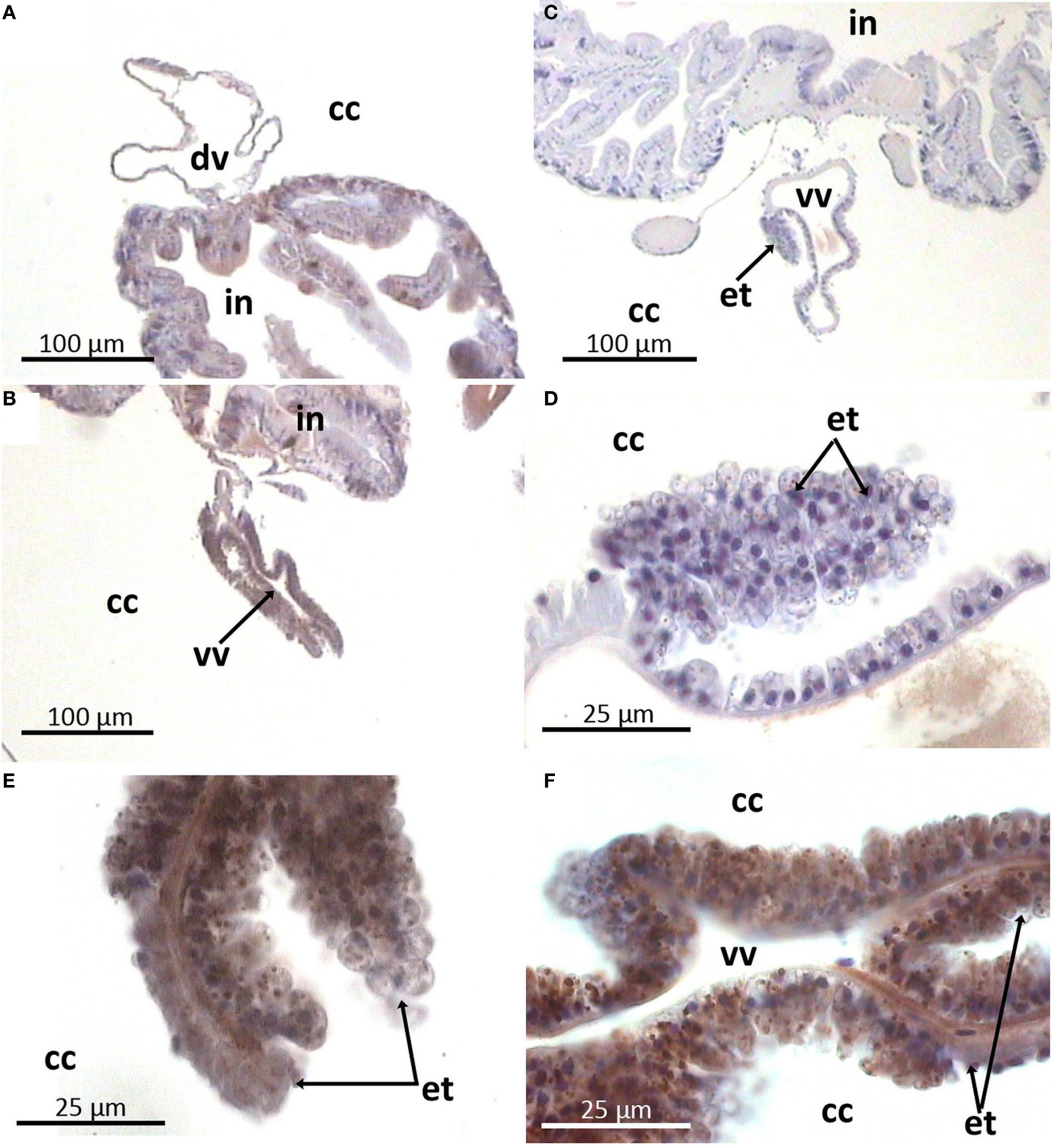
**Arenicin-positive immunoreactivity of lugworm vascular system**. **A,B**: general view of dorsal and ventral vessels respectively; **E,F**: extravasal tissue; **C,D**: negative controls; cc, coelomic cavity; dv, dorsal vessel; in, intestine; et, extravasal tissue; vv, ventral vessel.

In the gut wall arenicins were expressed only in sparse individual cells (Figure 6). These cells appeared to be intercalated into the epithelial layer and morphologically distinct from the others—they were larger in size and rounded as opposed to cylindrical enterocytes. Presumably these cells could be considered as a type of glandular cell (Figures 6D–F). In addition, high levels of arenicin expression were detected in tissues associated with ventral blood vessel in contrast to dorsal ones (Figures 7A–C). Usually this compartment is described as extravasal or chloragogue tissue (Figures 7D–F) (Gardiner, 1992). Coelomocytes were not visible on these sections.

### In *arenicola marina* coelomocytes arenicins are predominantly localized in the vesicular compartment

Next we examined the expression of arenicins in coelomocytes. AMPs translated from individual transcripts (in contrast to proteolytic fragments) usually include signal peptide and undergo co-translational transfer into the endoplasmic reticulum. This implicates their localization in the vesicular apparatus of the cell. In our previous study (Ovchinnikova et al., 2004) it was demonstrated that arenicins are synthesized as large precursors including pre-peptidic part. Here we wanted to test whether arenicins are stored in granules or other cellular compartments.

**Figure 8:**
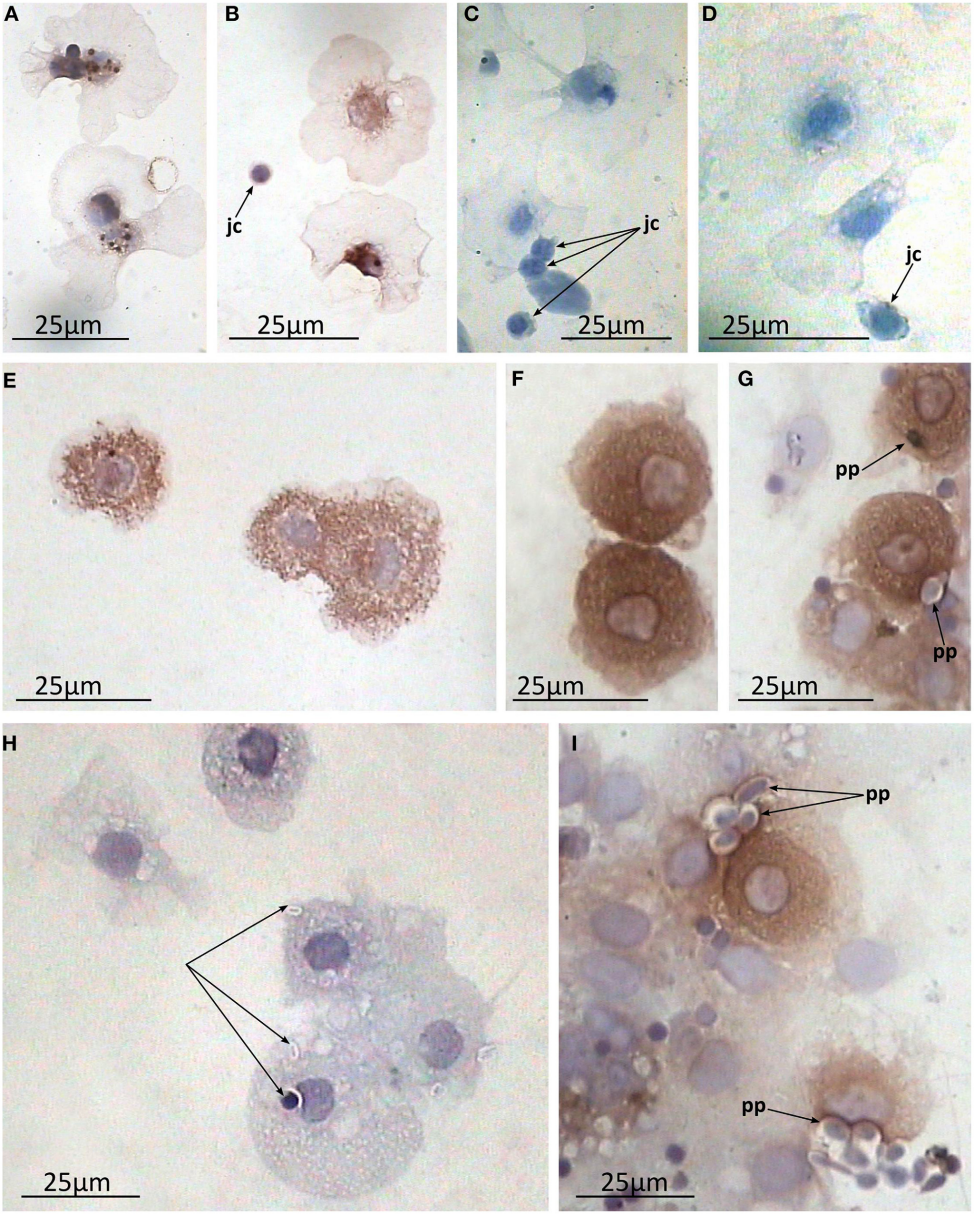
**Arenicin-positive immunoreactivity of lugworm coelomocytes**. **A–F**, coelomic fluid smears; **A,B**, granulocytes I and juvenile cells and **C,D**, corresponding negative controls; **E,F**, granulocytes II; **G–I**, *in vitro* phagocytosis preparations where **H**, negative control; jc, juvenile cells; pp, phagocytized particles.

Immunostaining of the preparations of the coelomic fluid smears demonstrated heterogeneity in the morphology of the coelomocyte population which supports previously reported observations (rev. in Vetvicka and Sima, 2009). Cells varied in morphology and the extent to which they spread on the substrate correlated with the number and size of the granules in their cytoplasm. Generally two main cell types were recognized on the smears. First were ones which possessed hyaline cytoplasm. They spread extensively and contained minimal large granules near the nucleus (granulocytes I, Figures 8A–D). The second type was characterized by numerous medium-sized granules filling up almost all of the cytoplasm (granulocytes II). Presumably, this well-developed granular apparatus prevented these cells from effective spreading, resulting in a more rounded form with just short and broad pseudopodia (Figures 8E,F). Additionally, putative juvenile cells (small cells with high nucleus / cytoplasm ratio without any granules) were also regularly observed as a minor part of the population (Figures 8B–D). Noteworthy, both types of mature granulocytes showed arenicins immunopositivity, whereas juvenile cells did not, suggesting that arenicins expression might be associated with maturation of coelomocytes. The arenicin signal was clearly associated with the granular apparatus of the cell, regardless of its organization.

### Coelomic fluid does not contain arenicins

AMPs stored in cytosolic granules can be liberated from cells via exocytosis. We investigated if arenicins could be found in coelomic fluid plasma (CFP). CFP was fractionated by HPLC and major fractions were analyzed by electrophoresis. Neat, as well as concentrated (1: 10) CFP appeared to be free from protein containing fractions with only one macromolecular component (MW > 10 kDa) in CFP detected. However, its detection was not regular. Notably, neither whole CFP nor any of its fractions showed detectable antimicrobial activity. Injection of microbes into coelomic cavity had no effect on the spectrum of CFP fractions as well as on the antimicrobial activity of CFP. Collectively, these results suggest that CFP does not contain anti-microbial components either under normal condition or after the microbial challenge.

### In the preparations of *in vitro* phagocytosis of lugworm coelomocytes arenicins are co-localized with the phagocytized particle

One of the main functions of AMPs is their participation in the process of microbial killing inside the phagolysosome. To test this possibility we immunostained the *in vitro* preparations of phagocytosis with anti-arenicin pAb. Both types of coelomocytes readily phagocytized available zymosan particles. In the individual arenicin-positive cells, engulfed particles were stained brown with a well visible brown rim surrounding them (Figures 8G–I), indicating the presence of arenicins within phagolysosomes. This suggests involvement of these peptides in the killing of phagocytized microbes.

## Discussion

In the present study we characterized the tissue distribution of previously described antimicrobial peptide arenicin, represented by two isoforms which differed by a single amino-acid replacement in the mature peptide (Ovchinnikova et al., 2004). Also arenicins-1 and -2 differ in 4 amino acids in the pro- and in 2 amino acids in the pre-peptide parts, respectively. It is still not ascertained as to whether these two peptides are the products of the same gene or of two individual genes. An unambiguous interpretation of the noticeable lapse in the PCR signal strength from two isoforms is not possible. However, as we did not reveal any other principal difference in their expression pattern (except signal strength) and as both isoforms were effectively recognized by the pAB, we will discuss them together.

There were three major findings in this study.

Firstly, arenicins are expressed in a wide range of tissues of the lugworm body in addition to coelomocytes where they were initially identified. The list of the tissues expressing arenicins includes several epithelia, which implies involvement of arenicins into both systemic and epithelial branches of immunity.

Secondly, expression of arenicins (resembling that of hedistin from *N. diversicolor*, Tasiemski et al., 2007) is not sensitive to the presence of different PAMPs in coelomic fluid.

Finally, although coelomocytes are capable of production and storage of arenicins, their secretion into coelomic fluid was negligible. However, our study did demonstrate that arenicins participate in the pathogen inactivation within the phagolysosome.

Lugworms are common dwellers in muddy habitats of subtidal and intertidal zones. Habitats such as these are abundant in microbes. Lugworms spend their lives engulfing and disgorging the surrounding sediments. Both epithelia—body wall and the gut—are under permanent threat of being attacked by microbial pathogens. Our findings supported this as we detected the expression of arenicins using both PCR and immunohistochemical methods in both critical organism boundaries—the body wall and gut. This characterizes arenicins as key players in the epithelial defense.

The outermost part of the body wall which was positive for arenicin consists of a thin cuticle and one layered epithelium. The principal cell type constituting the surface epithelium in polychaetes is represented by so called “supporting” cells. These cells bear apical microvilli which penetrate cuticle and are responsible for its synthesis (rev. in Gardiner, 1992). Generally cuticular components include proteins embedded into carbohydrate scaffolds (rev. in Gardiner, 1992). Immunopositivity for arenicins was detected in both the cuticle and the underlying cellular layer. It was also observed inside the chaetae, which are cuticular derivates. These data suggest that arenicins are implicated in protection of the outer boundary of worm body, alongside with the other proteins which are synthesized and secreted into the cuticle by supporting cells. Likewise, an AMP from oligochaete *Pheretima tshiliensis* (soil living worm)—PP-1, was immunodetected in the mucus, covering the body (although it was not detected in epidermal cells) (Wang et al., 2003).

Midventrally situated neuronal cord, lying upon circular musculature of the body wall, also proved to be arenicin-positive. The structure of the *A. marina* nervous system is not comprehensively characterized yet. In general, in polychaetes the outermost part of both segmentary ganglia and intersegmentary connectives is represented by projections of glial supporting cells. These projections span outside the cord and into it to provide mechanical support against muscular compression (Golding, 1992). In *A. marina* segmentary ganglia are not differentiated at all—neurons are evenly spread laterally along the ventral nervous chain (Wells, 1950). This is very well observed in negative control preparations (Figure 5A). Outer parts of the neural cord (an envelope and basolateral sectors) showed strongest signal of arenicin presence. It is possible that glial cells or neurons or both express arenicins inside the neural cord. The expression of two AMPs by glia and neurons was demonstrated in the medicinal leech *Hirudo medicinalis*. The expression was up-regulated in response to injury and microbial challenge implicating the involvement of AMPs in both defense and regeneration (Schikorski et al., 2008). The complexity and the degree of differentiation of the polychaetes ventral nerve cord never reaches the magnitude found in leeches (Golding, 1992). *A. marina* is accepted as an organism, which is incapable of regenerating any part of its body (Bely, 2006). Additionally, there is no reason to suspect the secretion of arenicins into coelomic cavity (see below). All these facts preclude direct extrapolation of revealed AMPs neuronal function in leech to lugworm. The functioning of arenicins in the nervous system still remains to be elucidated although their presence in this compartment indirectly supports the multifunctional nature of AMPs *in vivo*.

The gut is the second principal compartment of the lugworm as it exists in permanent contact with the surrounding mud. Interestingly, our study revealed that arenicins are also expressed in this area. The polychaete midgut usually consist of enterocytes as a dominant population and one or several types of secretory gland cells (rev. Saulnier-Michel, 1992). *A. marina* midgut contains both cell types (Kaganovskaya, 1978). Arenicin-positive cells are scattered irregularly in the epithelial layer and morphologically resemble gland cells. This implies that arenicins may be secreted into the gut lumen, although this requires further investigation. Similarly, AMPs of another annelid worm—theromicin and theromyzin from a leech *Theromyzon tessulatum*—were shown to be expressed both in intestinal and epidermal cells (Tasiemski et al., 2004).

As in original study (Ovchinnikova et al., 2004) it was reported that arenicins were isolated from coelomocytes, hence we expected to detect their expression in these cells. We tested if the level of arenicins mRNA expression is sensitive to different microbial stimuli by Real-Time PCR. To capture the spectrum of potential PAMP/PRRs interactions which may be involved in the regulation of arenicins expression we used three different heat-inactivated microbial strains (Gram-positive *L. monocytogenes*, gram-negative *E. coli* and fungi *C. albicans*) representing the principal groups of microbial pathogens. Notably, there was no difference in the levels of arenicin-1,2 expression upon challenge with any of the above stimuli, including combined stimulation, compared to non-stimulated controls. This suggests that arenicins expression is constitutive and could be explained by the nature of lugworm biology, in that all tissues examined in this study are continually exposed to the surrounding environment. As a consequence they have developed a certain degree of tolerance to various microbiota. In such a scenario constitutive expression would be more beneficial in contrast to inducible (as this was documented for some AMPs in epithelia of salivary glands and the ejaculatory duct in insects, Ryu et al., 2004).

We further addressed the functional role of arenicins as components of lugworm host defense. We examined the possibility for arenicins to be released into the CF thus providing the first line of defense acting as soluble factors. However, thorough analysis of CF by different biochemical methods did not reveal any traces of arenicins. Furthermore, the analysis of CFP antimicrobial activity demonstrated negative results, suggesting that in coelomic cavity, anti-microbial defense predominantly depends on cell-mediated immunity. Next we proposed that the main function of arenicins would facilitate the process of microbial killing within the phagolysosome. Cytoplasm of *A. marina* coelomocytes demonstrated well developed vesicular apparatus strongly positive for arenicins indicating that peptides are being stored in the vesicular compartment.

Generally there are several types of coelomocytes in polychaetes, such as granulocytes (syn. amoebocytes), eleocytes and hemocytes. However, the last two are accepted to be absent in Arenicolidae (rev. in Vetvicka and Sima, 2009). Granulocytes (amoebocytes) are further subdivided into several subtypes, although relationships between those are not well understood. In *Arenicola* these subtypes vary in their extent of development of granular apparatus and actin fiber networks (Chaga et al., 1998). Chaga et al. separated the terms amoebocytes (few granules, well developed actin fibers) and granulocytes (numerous granules, thin membrane associated actin network), which corresponded to granulocytes of types I and II respectively in Dhainaut and Porchet-Henneré, nomenclature (Dhainaut and Porchet-Henneré, 1988). Both cell types originate from the same source which are termed “juvenile cells” (Persinina and Chaga, 1998). Our data support this concept—granulocytes of both types are clearly recognizable and even their morphology differs from described previously (as they are actively spread on smears) as well as juvenile cells. Importantly, both granulocyte types contain arenicins in their granules unlike juvenile cells, which have no developed granular apparatus. This implies that arenicins expression in coelomocytes correlates with the maturation of their granular apparatus.

Still there is unambiguous interpretation of an interrelation among their subpopulations. Moreover, an evident demonstration of the common hematopoietic area is also lacking. Different derivatives of coelomic peritoneum including extravasal tissue are most often proposed as candidates (rev. in Gardiner, 1992; Vetvicka and Sima, 2009). Our data are in agreement with this suggestion. Extravasal tissue (a derivative of coelothelium) was surmised for hematopoietic activity (rev. in Gardiner, 1992). Its cells possess granular cytoplasm and participate in clearance of coelomic cavity via phagocytosis (Braunbeck and Dales, 1984, 1985). In the present study these cells along with coelomocytes were detected as producers of arenicins. That finding may suggest potential lineal relation between extravasal tissue and coelomocytes (as both populations were shown to possess arenicin positive granules and be able to phagocytize). The fact that arenicins were absent in CF and abundant in coelomocytes granules allows speculation about the role of arenicins in the killing of phagocytized pathogens. Even though we have not demonstrated involvement of arenicins in phagocytosis in extravasal tissue, we have shown this in the case of coelomocytes.

In granulocytes, immunopositivity was detected in granular apparatus, which was expected, as pre-pro-arenicins include signal peptides. There are two main destinations for cytosolic granules—to be exocytized or to fuse with the other vacuole. Immunohistochemistry of *in vitro* induced phagocytosis of *A. marina* coelomocytes demonstrated the co-localization of arenicins with the internalized particles suggesting involvement of arenicins in the process of pathogen inactivation (Figures 8G–I). To our knowledge this is the first report to demonstrate the role of AMP in phagocytosis within Annelids.

Coelomocytes were tested for induction of the arenicin gene upon infectious conditions and showed no change in arenicin expression. This result of constitutive arenicins synthesis in coelomocytes is concurrent with that of hedistin—an AMP constitutively expressed in coelomocytes of a benthic polychaete *Nereis decersicolor* (Tasiemski et al., 2007). Unlike *A. marina* where coelomocytes are the principal but not exclusive site of arenicins expression, in *N. diversicolor* the only one amoebocyte population—NK cells or granulocytes type 3—constitutively expresses hedistin. *Nereis* NK cells possess minimal large granules in the cytoplasm where hedistin is likely stored as its transcript encodes for a signal peptide. These cells are not phagocytic, but cytotoxic. They are able to actively migrate to the site of infectious invasion and release their content of granules. There are no NK-like cells in *Arenicola*. Despite both arenicin and hedistin being constitutively expressed in coelomocytes, their functioning in the worm organism differs.

Active phagocytic cells and epithelia are the most common compartments of AMP expression in metazoans (including mammals and insects). Interestingly, in the present study we revealed expression of the same peptide in both compartments. Usually epithelia and phagocytes produce their own set of AMPs (e.g., in humans—alpha-defensins 1-4 in neutrophils, LL-37 in monocytes, alpha-defensins 5-6 in intestine and different beta-defensins in skin and other borders) (De Smet and Contreras, 2005). Another remarkable fact is that there is no detectable antimicrobial activity in coelomic fluid, whereas in other investigated annelids (*Thremyzon* or *Nereis*) AMPs are secreted by producing cells to participate in host defense as humoral factors (Tasiemski et al., 2004, 2007). Signaling cascades, regulating AMP expression are thoroughly investigated in mammals and insects, but still unexplored in annelids. It is an urgent problem to clarify whether or not variability in AMP structure and functioning in annelids correlates with diversity or alternatively conservation of regulatory mechanisms. In this study we did not address the molecular mechanism of regulation of arenicin expression and this is a subject for future studies.

## Conclusion

Our study demonstrated that arencins are found in the tissues of the lugworm body (coelomocytes, body wall, extravasal tissue and the gut) which provide the first line of defense against infections. This supports the important role of arenicins as key components of both epithelial and systemic branches of host defense. It was established that expression of arenicins is constitutive and does not depend on stimulation of various infectious stimuli. In coelomocytes, arenicins function as killing agents inside the phagolysosome, and may potentially carry this trait in extravasal tissue. In the gut and body wall epithelia, arenicins are released from producing cells via secretion as they are found in the content of the cuticle and inside the midgut gland cells. This study demonstrates that distribution and functioning of arenicins in the lugworm share some features with AMPs of other annelids but overall their characteristics are quite unique.

## Conflict of interest statement

The authors declare that the research was conducted in the absence of any commercial or financial relationships that could be construed as a potential conflict of interest.
