# Supplementary material for: Expression pattern of arenicins—the antimicrobial peptides of polychaete Arenicola marina
Source: Front Physiol. 2014 Dec 19;5:497. doi: 10.3389/fphys.2014.00497 (PMC4271772; doi:10.3389/fphys.2014.00497)
Supplement: Supplementary file 1 [file DataSheet1.DOCX]

**Supplementary figure captions.**

**S1. mRNA expression levels of arenicin-1 and -2 (A) relatively to actin (B) measured by PCR.** Expression levels were measured in coelomocytes (1, 2), body wall (3, 4), pharynx (5, 6) and intestine (7). Arinicin-1 PCR product is in the left lane and arenicin-2 - in right one in each pair, both amplified on the same matrix. Only one actin control was made for each pair, because an equal quantity of cDNA was used for amplification of the both arenicins.

**S2. mRNA expression levels of arenicin-1 measured by RT-PCR relatively to actin after immunization in the body wall (A) or intestine (B).** Microbial mixture 24h post infection (1), *C*.*albicans* 24h post infection (2), *E.coli* 48h (3) and 24h post infection (4), *L.monocytogenes* 24h post infection (5), PBS 24h post infection (6), intact animals (7). Data are expressed as mean±SD.
