# Supplementary figures and images for: Expression pattern of arenicins—the antimicrobial peptides of polychaete Arenicola marina
Source: Front Physiol. 2014 Dec 19;5:497. doi: 10.3389/fphys.2014.00497 (PMC4271772; doi:10.3389/fphys.2014.00497)

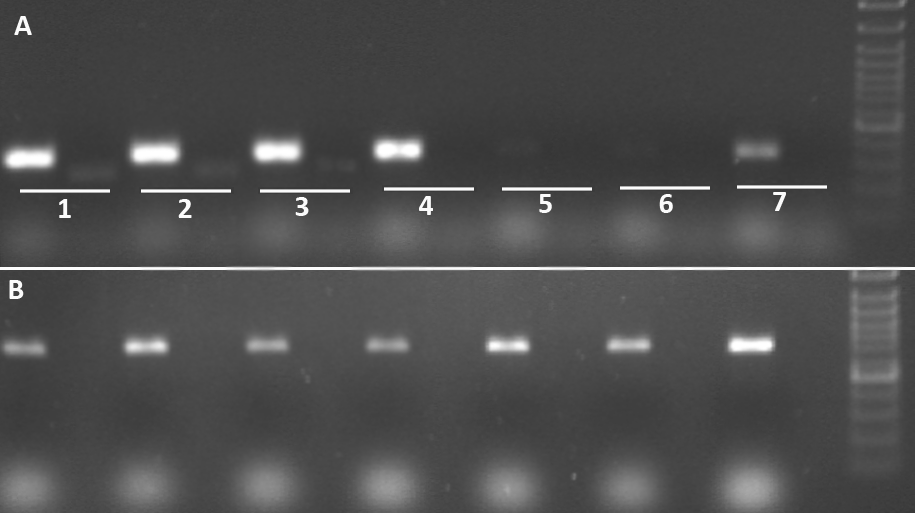

Supplement: Supplementary file 2 [file Image1.TIF]

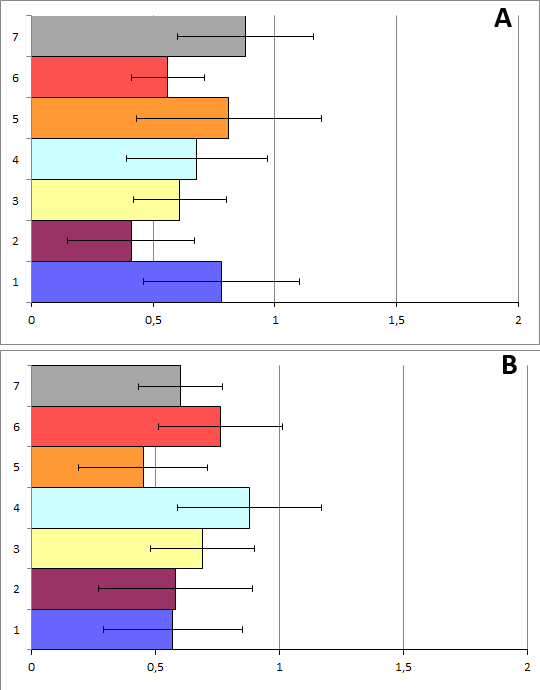

Supplement: Supplementary file 3 [file Image2.TIF]
